# Treatment costs and priority setting in health care: A qualitative study

**DOI:** 10.1186/1743-8462-6-11

**Published:** 2009-05-06

**Authors:** John McKie, Bradley Shrimpton, Jeff Richardson, Rosalind Hurworth

**Affiliations:** 1 Centre for Health Economics, Faculty of Business and Economics, Monash University, Clayton, Victoria 3800, Australia; 2 Centre for Program Evaluation, the University of Melbourne, Parkville, Victoria 3010, Australia

## Abstract

**Background:**

The aim of this study is to investigate whether the public believes high cost patients should be a lower priority for public health care than low cost patients, other things being equal, in order to maximise health gains from the health budget. Semi-structured group discussions were used to help participants reflect critically upon their own views and gain exposure to alternative views, and in this way elicit underlying values rather than unreflective preferences. Participants were given two main tasks: first, to select from among three general principles for setting health care priorities the one that comes closest to their own views; second, to allocate a limited hospital budget between two groups of imaginary patients. Forty-one people, varying in age, occupation, income and education level, participated in a total of six group discussions with each group comprising between six and eight people.

**Results:**

After discussion and deliberation, 30 participants rejected the most cost-effective principle for setting priorities, citing reasons such as 'moral values' and 'a personal belief that we shouldn't discriminate'. Only three participants chose to allocate the entire hospital budget to the low cost patients. Reasons for allocating some money to inefficient (high cost) patients included 'fairness' and the desire to give all patients a 'chance'.

**Conclusion:**

Participants rejected a single-minded focus on efficiency – maximising health gains – when setting priorities in health care. There was a concern to avoid strategies that deny patients all hope of treatment, and a willingness to sacrifice health gains for a 'fair' public health system.

## Introduction

To assist decision-makers in allocating resources between different health programs and services economists use cost-benefit analysis or cost-effectiveness analysis. What they have in common is the central role they assign to costs. For economists, patients who can be treated at a low cost should always have priority over high-cost patients, other things being equal, since this allows more patients to be helped in the context of a limited budget [1,2]. This reflects the utilitarian foundations of modern economics – limited resources should be used to achieve the maximum amount of good, where 'good' in this context means improvements in the quality and/or length of life. However, studies in Australia, Spain and Canada (detailed in the next section) provide evidence that the populations in those countries reject a single-minded focus on maximising health for each dollar spent.

The present paper reports the results of a study of the Australian public's views on this issue. It sought, in particular, to elicit reflective, considered views on the relevance of treatment costs, and to explore the values, arguments and rationalisations underlying those views. This was achieved by using semi-structured group discussions that involve dynamic and rich group conversations in which individuals present ideas, hear from other participants and can question each other [3,4]. To our knowledge it is the first study of public attitudes towards treatment costs to use such deliberative methods.

### Previous empirical studies

Finding out what the public thinks is, or should be, of central concern to economists, since it is not their role to impose their values on the public [5]. It is therefore surprising there have been so few empirical studies of social attitudes towards treatment costs. In one of the few empirical studies in this area, individuals surveyed in Australia in 1995 clearly rejected the idea that health services should be provided on the basis of least cost [6]. In stage 1 of the study, which used a postal questionnaire, subjects were asked whether (1) those who can be helped at low cost should have priority over those who can be helped at high cost, or (2) whether it is unfair to discriminate against those who happen to have a high-cost illness, except in cases where costs are extremely high. Only 18.6% of those surveyed were in favour of giving priority to low-cost patients, while 81.4% thought that high- and low-cost patients should be treated equally. One group of subjects was given a slightly re-worded version of option 1 that replaced the words 'should have priority' with the weaker 'should have some priority'. A clear majority of 70% still chose option 2.

In stage 2 of the study, some participants were interviewed and asked about their reasons. When those in favour of the equal priority option were challenged about their position they emphasised that people cannot be blamed for getting high-cost illnesses, that other factors such as severity of illness are important, and that everyone is entitled to treatment irrespective of cost [[6], p. 85]. According to the authors, the results suggest that:

The concern with allocative efficiency, as usually envisaged by the economists, is not shared by the general public and that the cost-effectiveness approach to assigning priorities in health care may be imposing an excessively simple value system upon resource allocation decision-making [[6], p. 79].

In a Spanish study, Abellan-Perpiñán and Prades [7] asked subjects to allocate a budget between two patients. It would cost one million pesetas to extend the life of John for one year and two million pesetas to extend the life of Andrew for one year. When asked how they would split the money, 74% chose to ensure both patients would gain 16.6 years, although this meant spending twice as much on Andrew (33.3 million pesetas) as on John (16.6 million pesetas). Only 4% were willing to spend more on John than on Andrew, even though this was the most cost-effective alternative. When the same subjects were asked to imagine that Andrew would live the rest of his life undergoing dialysis, 84% did not change their view. In a separate exercise (Group 2), subjects were told from the beginning that Andrew would live the rest of his life undergoing dialysis. In this case only 29% chose to ensure that both Andrew and John would gain 16.6 years (by allocating 33.3 million pesetas to Andrew and 16.6 million pesetas to John). However, the support for health maximisation was still weak, with only 27% of subjects being prepared to spend more on John than on Andrew, although this was the most cost-effective alternative.

Finally, in a Canadian study by Abelson, Lomas et al. [8], participants were asked to rank the following in terms of their importance for health care and social service decision-making: needs; benefits; costs; preferences. Five different groups took part in the study – randomly selected citizens, attendees at town hall meetings, appointees to district health councils, elected officials and experts in health care and social services. Costs were rated third in importance by all groups except the elected officials, who placed it second. All five groups thought that information about needs was most important, with benefits being rated second by all groups except the elected officials. Information about preferences was placed last by all groups.

## Methods

To explore the public's values and reasons for attaching less significance to costs than most economists would consider appropriate, the present study utilised group discussions, or what are also commonly called 'focus groups'. Research theorists, including Ritchie [9] and Finch & Lewis [3], and others, have noted their preference for the term 'group discussions', arguing that it more clearly identifies that when using this method 'data is generated and shaped through discussion' [[9] p. 37]. For this reason we refer to 'discussion groups' throughout this paper.

### (1) The two main tasks

The study took place in Victoria, Australia, with 41 people participating in a total of six group discussions with each group consisting of between six and eight people. Informed consent was obtained from all participants and the study was approved by the Monash University Human Research Ethics Committee. On average, sessions ran for 1 to 1.5 hours. Initially, each discussion began with a warm-up question that asked participants to provide ideas on how they would advise the government to allocate its health budget on the assumption that it is limited and under increasing pressure, and to suggest the issues these decisions might raise.

This was followed by two distinct stages in the group discussions. In the first stage, participants were presented with three principles for allocating health resources among patients who are equally ill, but who have different costs associated with their treatment. The first and third principles were derived from the earlier Australian study [6]:

• Alternative 1: Among patients who are equally ill, those who can be helped at low cost should have priority over those who can be helped at high cost, because this will allow more people to be helped when money is limited.

• Alternative 2: It is unfair to discriminate against those who happen to have a high-cost illness. Priority should therefore not depend on the cost of treatment.

• Alternative 3: It is unfair to discriminate against those who happen to have a high-cost illness. Priority should therefore not depend on the cost of treatment – except in cases where costs are extremely high.

These options were projected onto a screen using a computer and data projector, so that participants could engage more easily in discussion without having to remember the details of each alternative. Participants were encouraged to debate the merits and demerits of decisions based on each alternative, and at the conclusion of this part of the task were asked to indicate which of the options came closest to their own views.

The second stage involved participants engaging with the same type of choice, but this time presented in a different form. Respondents were asked to imagine they were members of a hospital committee responsible for allocating a budget between two groups of equally ill patients. The cost of treating each patient in the first group (Disease 1) was $40,000, while each patient in the second group (Disease 2) would cost $20,000. Again using a data projector, participants were shown eight ways a budget of $12 million might be distributed among the two patient groups and the benefits and consequences – in terms of total patients treated or not treated – associated with each option. This second stage was undertaken as a triangulation exercise to explore if participant responses were likely to change if they encountered cost considerations in different ways, in particular if the 'opportunity cost' of each option was made very clear and the range of options expanded. This is described in more detail in the Results section that outlines the study's findings.

### (2) Composition and conduct of the groups

To include a variety of perspectives, a range of participants were recruited for the group discussions. Participants were selected purposively to include a spectrum of ages, an assortment of occupational groups, people with different levels of income and educational backgrounds. While sampling was considered carefully, our study was exploratory, with the aim of detecting different viewpoints held in the community, but not on making generalisations to larger populations, and the results should not be viewed in this way. Having a diverse range of participants ensures that the groups are not unrepresentative, but does not ensure that they are representative. For the latter a much larger sample is needed.

We identified key informants associated with occupational and other groups – for example, building maintenance workers, social workers, and students studying to be teachers (see Table 1) – and through these key informants invited their colleagues or co-workers to participate in the study. There are various arguments for choosing to use either heterogeneous or homogeneous discussion groups (for a discussion of this topic see Hesse-Biber and Leavy [4]). However, following the advice of Finch and Lewis [3] we decided to use largely homogeneous groups as these types of groups: increase the likelihood of group cohesion through participants having experiences in common; reduce the possibility of imbalances in power or social status that can inhibit discussion; and bring to the fore the degree to which differently constructed groups hold different perspectives. At the same time, we encouraged individuals to put forward their own points of view and not to feel compelled to agree with the views of others.

**Table 1:** Composition of the discussion groups

Group	Number of Participants	Characteristics
DG 1	6	Social workers
DG 2	6	Middle-aged professionals
DG 3	7	University administrative staff
DG 4	8	Second and third year education students
DG 5	7	Tradesmen and building maintenance workers
DG 6	7	University administrative staff

Groups were moderated by 'complementary moderators' [10] consisting of a member of the research team with particular expertise in the facilitation of group discussions, and a second moderator with extensive knowledge of health economics who could answer participant questions and provide additional information to assist deliberations. The six groups were tape-recorded with all tapes transcribed in full. Transcripts were then coded, beginning with a basic set of codes established through a review of the literature. Codes were maintained, adapted, added to or collapsed following further close readings of the text. Next, all major ideas for each group were displayed under thematic headings on matrices of the type proposed by Miles and Huberman [11]. These displays, in combination with verbatim quotes from the transcripts, are a particularly rigorous way of dealing with such qualitative data. This process revealed the major themes, common perspectives and differences, on the role of costs when setting health priorities, which emerged across the six groups.

## Results

### (1) Participant responses to priority setting possibilities

As noted above, we began the group discussions by first showing participants three possible principles or options that might be used to guide resource allocation among patients (alternatives 1 to 3 above). At the conclusion of this, members of each group were asked to indicate which of the alternatives came closest to their own views. In this section we overview the various positions taken by participants when presented with each alternative, and the reasonings and arguments used to justify or reject them.

#### (a) Alternative 1: Low-cost patients should have priority

Only six participants from all groups initially agreed that among patients who are equally ill those who can be helped at a low cost should have priority over high-cost patients. While one respondent felt that favouring the majority (low cost) over the minority (high cost) was a '*fairer*' decision that created the opportunity of '*doing more good*', others indicated they only reluctantly selected this as being the '*lesser of evils*' and a better choice among ultimately '*discriminatory*' alternatives. Two participants also added that some money should still be given to high-cost patients '*in order to be seen to be doing something for all parties*' (DG 3).

The majority of participants, however, typically fell into one of two categories. The first grouping, consisting of approximately two-thirds of respondents, rejected outright prioritising low cost patients as '*unfair*'. Even when it was pointed out that fewer patients would be treated if this alternative was not adopted these participants were unmoved and defended their position with statements such as: "But I think that accepts that money is the bottom line in health care. I won't accept that..." (DG 1). As a possible way forward some participants suggested that health spending could be "distributed fairly (equally) among both (high-cost and low-cost groups)" (DG 5). This would still mean that more low-cost patients would receive treatment since they are cheaper to treat, but it does not require abandoning high-cost patients altogether. People in three groups who had taken this position additionally proposed that any shortfalls in treatment costs would need to be met by patients themselves, or be partially contributed to by patients if they were low income earners (DG 2, 3, 5). Meanwhile, one participant felt that means-testing might also be an option arguing that "if you could afford to cover your costs then maybe you should have to" (DG 3). Taking a different approach altogether several respondents proposed that treatment should be given on a first come, first served basis (DG 4).

At this initial stage of the discussions the remaining type of response came from participants who, despite being asked to consider the patients 'equally ill' – indeed, the same in all respects except treatment cost – searched for mitigating factors that could override the need to make a judgement based on costs and sheer numbers. Participants in two groups held strong opinions that issues of lifestyle (e.g. smoking) needed to be taken into consideration when making allocation decisions (DG 1, 4). Others wanted to know the recovery prospects of patients, with some participants willing to direct treatment to those whose quality of life would be highest after treatment (DG 2, 3). Last, for one young, and another not so young participant, age was a determining factor when allocating resources, with both willing to favour the treatment of younger patients before older patients. This second group is discussed further below.

#### (b) Alternative 2: Priority should not depend on cost

Given the obvious discomfort that the first alternative caused some participants, and the general reluctance of most to prioritise low-cost patients, it was perhaps surprising there was not a groundswell of approval for abandoning cost altogether as a criterion. Indeed, possibly reflecting the accumulative effect of deliberation, participant opinions began to shift noticeably. When asked if prioritisation should not take into account treatment costs at all participants again largely fell into two groups, but now numbers were roughly even.

In the first group were those who enthusiastically embraced the idea that priority should not depend on cost and that resources should be "distributed evenly among both (high-cost and low-cost patients)" (DG 5). Participants said this alternative was "fairer ... because you don't choose to get a high-cost illness" (DG 4) and pointed out that, as taxpayers, citizens should "expect to be looked after by the public health system..." (DG 4). Others indicated that this alternative sat more easily with personal beliefs that an individual's life should not be privileged or penalised on the basis of cost (DG 1), with one participant explaining that "just because you're high cost doesn't mean you go to the bottom of the list" (DG 3). Meanwhile, comments from several participants placed them in the 'denial' category identified above – they were unwilling to accept the need for cost-based rationing despite Australian newspapers and other popular media routinely reporting a looming health crisis due to scarce health resources [12].

In the second grouping were quite a large number of participants who, like those in the first grouping, felt that decisions based on costs were "unfair" (DG 2), "unpalatable" (DG 3), and caused some participants more "discomfort" (DG 4), but who were now prepared to accept that costs should play a role in health care prioritisation. Summing up the conflict experienced by many, one participant remarked, "I think it is unfair, but we should (be mindful of costs). That's why society must change" (DG 4). Participants in this second grouping discussed how "there is a limited pot" (DG 2) of money for health care, and that treating low-cost patients at the expense of higher-cost patients would mean that "at the end of the day you are treating more" (DG 6). Others also saw this choice as potentially achieving greater good for the community, explaining that by treating more patients, well patients could then "go out and help make more money for the system" (DG3). While willing to consider costs in treatment decisions there were nevertheless strong feelings among many in this second grouping that life should not be seen as "easily expendable" (DG 1), with calls for arrangements that ensured some proportion of high-cost patients were treated or were allocated a share of health resources (DG 6). This issue was also raised in discussion of the final alternative.

#### (c) Alternative 3: Extremely high-cost treatments

The prospect of not allocating resources to 'extremely high-cost' patients at first generated emotional responses from participants in most discussion groups. Group members described friends, relatives or their own experiences of battling difficult and expensive illnesses. However, taken as a whole, while participants again found this alternative "not the most palatable" (DG 6), few participants rejected it. According to one participant there needed to be "... a line where you say 'right, anything over that cost is extremely high (and won't be funded)' " (DG 5), a position only a handful of participants were unwilling to accept. Instead, the focus of participant discussions for the most part centred on two matters.

The first of these arose in three discussion groups (DG 1, 2, 5) where respondents were prepared to accept that resources should not be directed to extremely high-cost patients, but with some exceptions. Returning to arguments previously put forward during discussion of Alternative 1, participants in these groups felt that age should be a factor when choosing or excluding patients from receiving extremely costly treatments. In an expression of 'ageist' preferencing, one of these participants suggested that costly treatments might be funded for "a small child ... (but not) ... people of 80" (DG 1). This idea was discussed further by a second group where respondents felt that it was important to consider the length of time a young person would benefit from treatment as compared with older patients (DG 5). Meanwhile, a participant in a third discussion group acknowledged that her wish to treat the young regardless of cost came from an "emotional perspective", and explained that "I wouldn't tell a child that we can't give them support ... as a mother I wouldn't want someone to tell me that" (DG 2).

The second matter that received attention, which focused squarely on the issue of cost, was the desire not to withhold some form of treatment, or at least the allocation of some health resources, from costly patients (DG 2, 4, 5). For example, one participant was prepared to accept that scarce resources should not be directed to extremely high-cost patients but argued that "some funds need to be available to make their quality of life better ... at least to make them comfortable" (DG 2). Another participant felt that patients should receive extremely high-cost treatments if their condition "was life threatening" (DG 6). Others proposed that some form of funding threshold should exist after which – recalling suggestions offered during discussions of Alternative 1 – high-cost patients would need to self-finance any monetary gap in order to receive extremely expensive treatments (DG 4). Finally, as with Alternative 1, there were again suggestions (although this time from a different discussion group) that means-testing might be used, requiring those who could contribute to the cost of very expensive treatments to do so (DG 5).

#### (d) Final positions

Having been presented with, and discussed in detail, three possible ways that resources might be allocated among patients, participants were lastly asked to indicate which of the three alternatives came closest to their own beliefs. A number of participants found this task quite perplexing, describing an inner tension between "ideal" or "utopian" solutions (DG 3) and the need to be "objective" (DG 2) and face "reality" (DG 3). However, noting the need to "look at the money, not emotions" (DG 1) and the desire to "help as many people as you can" (DG 2) nine participants ultimately selected Alternative 1, that low-cost patients should be prioritised (see Table 2). Taking a very different stance, 15 participants cited reasons such as "moral values" (DG 4) and "a personal belief that we shouldn't discriminate" (DG 3) in justification of their preference for Alternative 2, that priority should not depend on the cost of treatment. Seeking a form of "balance" (FG 4), 15 participants selected Alternative 3 as their preferred approach to treatment costs. Last, two participants found they were unable to make a definite choice between the three options.

**Table 2:** Number of participants favouring the three allocation principles: Final positions

Alternative	No
**1**. Low cost patients should have priority	9
**2**. Priority should not depend on cost	15
**3**. Only extremely high cost treatments relevant	15
Unable to choose	2

Total	41

### (2) Participant responses to allocating a hospital budget

To gauge if participant's prioritisation choices would change if they encountered different hypothetical cost scenarios, discussion groups were next invited to imagine they were members of a hospital committee responsible for allocating a budget of 12 million dollars. The primary purpose of this exercise was to ensure that participants were absolutely clear about the consequences of their choices. Participants were asked to distribute the money between patients with two similar debilitating diseases. While the two (un-named) diseases were described as alike in most ways, participants were told that Disease 1 would cost twice as much to treat as Disease 2. Faced with eight possible strategies for distributing the 12 million dollars (see Table 3), participants made the following choices and offered the following explanations:

**Table 3:** Options for allocating a hospital budget

Number Treated	Options							
	1	2	3	4	5	6	7	8
	(All money for low cost patients)			(Same money for both diseases)	(Same number of patients)			(First come, first served)
Disease 1 (Cost/patient $40,000)	0	50	100	150	200	250	300	?
Disease 2 (Cost/patient $20,000)	600	500	400	300	200	100	0	?
**Total Treated**	**600**	**550**	**500**	**450**	**400**	**350**	**300**	**Approx 400**
**Number Not Treated**	**600**	**650**	**700**	**750**	**800**	**850**	**900**	**Approx 800**
Total Cost	12 m	12 m	12 m	12 m	12 m	12 m	12 m	12 m

#### (a) Option 1: The efficiency solution

This option was chosen by just three participants who indicated they believed it was "fairer to treat the most people possible" (DG 3). Indeed, the words 'fair', 'fairer', 'fairest', or similar variations, were used by participants to justify the selection of all but one of the nominated options, and was used abundantly by the following participant when justifying their choice of Option 1:

If you don't get treated in general it's not fair to you. If you're not treated that's not fair. You know what I mean? So why not make it the least amount of people feeling it's not fair? (DG 4)

#### (b) Options 2 and 3: Compromise solutions

Seven participants selected option 2 and fourteen selected option 3. They felt these options offered a "fair go" (DG 5) ('fair go' is an Australian colloquial expression that roughly means equality of opportunity [13]), through treating high numbers of patients, keeping untreated patient numbers low, while also, importantly for these participants, not removing the possibility of high-cost patients receiving treatment. In contrast to those who selected Option 1, these participants were adamant that it was essential not to remove the "chance" (DG 5) of being treated or to "discriminate completely" (DG 3) against Disease 1 patients. People in two discussion groups additionally commented on the need to treat some Disease 1 patients in order to, "hopefully learn more about the disease and get the cost down ... so that your health budget is improving all the time" (DG 1).

#### (c) Option 4: Fiscal equity

Option 4 was nominated by eight participants as their preferred method for allocating the budget. This group of respondents argued that distributing the money in this way was "fairer" (DG 6) than the other options as it removed altogether cost-based prioritisation. While this option was the second most popular choice among participants, its selection by only eight participants (or a total of 16 when combined with those who nominated options 5 and 8) was consistent with the gradual move away from an initial refusal, at the beginning of the group discussions, to differentiate between patients based on treatment costs.

#### (d) Option 5: Patient group equity

Six participants selected option 5, with one emphatically declaring that "it is the only option" (DG 4). This strategy was selected as being more 'equitable' because it ensured that identical numbers of patients would be treated from both disease groups (DG 5), with participants willing to accept that "it's towards the worst case scenario (of untreated patient numbers) but doing anything else would be unfair" (DG 5). However, not all were rigidly committed to this alternative, with several acknowledging they were also drawn to option 4, and one participant said they would cease to support option 5 if the cost of treatment became excessive (DG 5).

#### (e) Option 8: First come, first served

Last, only two participants, from two different groups, felt that treating patients on a first come, first served basis was the best choice among the eight strategies. This option was selected as being closest to "what happens now... it's a competitive world out there" (DG 5), and was the only option where the word 'fair' was not used. No participants choose either option 6 or 7. One participant found they were unable to nominate a preferred strategy while another, summing up the feelings of many, remarked that, "I'm starting to see how difficult it is for people who make these decisions. We criticise them but they are very hard decisions" (DG 6).

## Discussion

Consistent with previous studies, respondents in the present study rejected the orthodox economic approach to costs. In the first stage of the study only six participants agreed that among patients who are equally ill those who can be helped at a low cost should have priority, with the majority finding this 'discriminatory' and 'unfair'. After discussion this reaction lessened and support for option 1 increased to nine, with many feeling torn between a commitment to fairness and the need for efficiency. In the second stage of the study, however, the number of participants supporting option 1 decreased to just three, with the majority favouring some form of compromise (trade-off). Two explanations for this change in the second stage are (i) the emphasis placed on exactly how many patients will not be treated on each option, and (ii) the greater range of alternatives on offer (increasing from three to eight).

**Figure 1:**
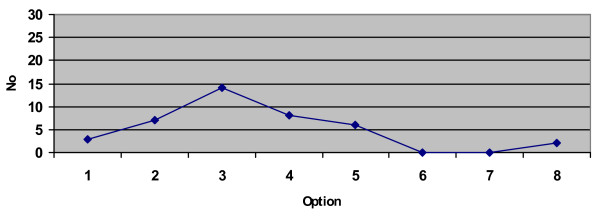
**Number of participants favouring the eight options for allocating a hospital budget (*N *= 40)**. Option 1 = All money for low cost patients. Option 4 = Same money for both diseases. Option 5 = Same number of patients. Option 8 = First come, first served.

Four of the eight strategies offered in stage 2 were governed by a clear principle – 'all the money for disease 2', 'the same amount of money for both diseases', 'the same number of patients' and 'first come, first served'. It is worth noting that these four options attracted less support than the 'no-name' option 3. Although the numbers are small, graphically they represent a hump-shaped curve with a single peak at option 3, suggesting it was not the laudable-sounding labels attached to some strategies that attracted respondents (see Figure 1). Rather, most situated themselves somewhere between the 'efficiency principle' (option 1) – 'efficiency' understood here as health maximisation – and the 'equity principles' (option 4 and above).

### (1) Participants' understanding of the task

It is worth noting that participants appeared to have no difficulty grasping the concept of economic cost. For economists, money per se is not the issue, it is the benefit forgone. In the first stage, for example, those who expressed a preference for the most cost-effective option did so on the ground that this would create the opportunity of 'doing more good' or because of a desire to 'help as many people as you can'. Similarly, participants discussed how treating low-cost patients at the expense of higher-cost patients would mean that 'at the end of the day you are treating more'. In the second stage, opportunity costs were actually quantified and the patients who would fail to be treated on each option were clearly shown. Again, those who chose the cost-effective option did so on the ground, for example, that it is 'fairer to treat *the most people possible*'. Although the term 'opportunity cost' might not have been used, it was the main topic of conversation, and was clearly understood by participants.

It might be thought that respondents *must have *failed to grasp the notion of opportunity cost or they would not have rejected the cost-effective alternative. That is, if the patients are alike in all respects, the only explanation for participants choosing to treat fewer rather than more patients is that they (are irrational or) failed to fully comprehend the implications of their choice; that is, they failed to understand the notion of opportunity cost. However, there are other explanations for respondents rejecting the cost-effective option, and these clearly came to the fore in the group discussions; for example, 'fairness', a desire not to 'abandon' people, a desire to give everyone a 'chance'. Judging by the reasons participants actually gave, it was not a failure to appreciate the concept of opportunity cost that led them to the reject the cost-effective option, but a willingness to trade-off health gains to secure these other goods.

Related to this, the fact that participants raised issues about health *benefits *does not mean they were off-target. For example, some respondents questioned not the issue of cost per se but rather the maximand; that is, some seemed to think the rule of rescue should apply (do not abandon small groups of high-cost patients). Meanwhile, others seemed to be arguing for some weighting of probabilities of outcomes (which might reflect a desire for using regret theory or prospect theory rather than expected utility theory), and many were interested in equity concerns and were thus questioning the distribution of health benefits rather than costs per se. However, the study was concerned with exploring the *reasons *for placing more or less emphasis on costs. It was a qualitative study that sought to know why (as previous studies had discovered) the public places less emphasis on costs than economists would deem appropriate; that is, what *other considerations *participants consider important, and for which they would be willing to trade-off overall health gains. Hence, it was entirely appropriate for participants to bring up issues such as the fairness of different distributions, health maximisation as an objective, the importance of not removing hope, the rule of rescue, and so on. It would be wrong to think that because participants raised these reasons for *downplaying the importance of costs*, that they were questioning something *other than *the relevance of cost.

Finally, it should be noted that the perspective adopted in the present study did not change between stage 1 and stage 2. In both stages participants were asked to give their *personal *views about a *social *issue. That is, the researchers were in effect saying to participants: "We want your *personal *view about how this *social *judgement – this judgement affecting different groups of hypothetical patients (with different costs associated with their treatment) – should be made". There was no change from stage 1 to stage 2 in this regard. In the second stage participants were asked to imagine they were members of a hospital committee responsible for allocating a budget. But this does not signal a change of perspective; for example, from the personal to the social. In both stages participants were asked to adopt what Nord calls a 'caring-for others perspective' [[14] pp. 8–12] – to make judgements about other people (i.e. hypothetical patients) – but to give their own personal view.

### (2) The preservation of hope

One of the main focuses of the study was on uncovering the reasons for participants' choices. In the second stage a number of participants expressed the view that it was essential not to remove the "chance" (DG 5) of being treated or to "discriminate completely" (DG 3) against Disease 1 patients. Some had strong feelings that life should not be seen as "easily expendable" (DG 1). In contrast to those who selected option 1, these respondents were concerned with ensuring that high-cost patients are not left in a 'hopeless' position. This is consistent with the results of two studies of organ transplantation undertaken by Ubel and Loewenstein [15]. The authors conducted a hypothetical 'organ allocation exercise' and found that participants wanted to allocate some organs to those with the worst prospects of survival. Participants explained their decision with comments such as "everyone deserves a chance" and "needy people deserve transplants, whatever their chance of survival" [[15] p. 1052] (see also [16-18]). To deny a person with a serious illness any hope of a cure, even when the probability is low, is to add an extra dimension of anguish to their remaining life over and above the suffering caused by the illness itself. A number of participants in the present study were prepared to sacrifice health gains to prevent this.

### (3) The 'essential contestability' of fairness

Throughout the discussions we saw participants grappling with the concept of fairness itself: in these cases it was not always a conflict between fairness and efficiency, but between fairness as treating the greatest number of people, versus treating the same number of people from both groups, versus giving the same amount of money to both groups. All of these, including the efficiency option, were seen as being fair in their different ways, with none having ethics unequivocally on its side. It is central to the concept of fairness that it involves treating people with "equal concern and respect" [[19] pp. 272–273], but different interpretations of what this means in practice are possible. In this sense fairness is an "essentially contestable" concept [20,21]; that is, while there is sufficient agreement on its basic meaning to enable discussion and debate, there is disagreement about the application of this concept in concrete cases – to actions, policies, institutions etc. This was evident in the group discussions reported here, where a range of incompatible options were justified on grounds of fairness with equal conviction. Interestingly, only the most 'arbitrary' option – first come, first served – was not explicitly defended on grounds of fairness.

### (4) Reluctance to trade

It was obvious that some participants found the conflict between the need to allocate resources efficiently and the ethical imperative to treat people equally very confronting. One response to this was to shift the focus onto other factors, such as the age of the patients, their smoking behaviour, or their different potentials for improvement. This was despite being asked to consider the patients 'equally ill' and 'alike in all other respects'. The moderators responded in this situation by asking participants which strategy they would adopt *if *there was the same number of smokers in each group, *if *there was the same distribution of ages, the same prospects for recovery, and so on. For some participants this had the effect of clarifying the task and bringing the discussion back to the issue of cost. For others the resistance remained. The moderators did not force these participants, recognising that refusing to accept the terms of the exercise, or rejecting the assumptions on which it is based (e.g. limited resources) are just alternative responses to the difficult choices participants were required to make [22,23]. However, while different participants exhibited different levels of resistance, the majority eventually came to the conclusion that there is a need to take some account of costs.

### (5) The importance of deliberation

Another central aim of the study was to afford participants the opportunity to deliberate and discuss the issue of treatment costs, and, in this way, to question a crucial assumption underlying the current practice of "implicit rationing" [24]; that is, minimise cost per unit of benefit. The course of each discussion group was similar – at the beginning, some people rejected the relevance of costs altogether, and others looked for ways of avoiding the need to make hard decisions. After discussion, however, most participants came to see this as "ideal" or "utopian" (DG 3) and began to recognise the need to take some account of cost – the need to be "objective" (DG 2) and face "reality" (DG 3). We are therefore inclined to agree with Payne, Bettman et al. [25], who argue that preference elicitation is best viewed "as architecture (building a set of values) rather than as archaeology (uncovering existing values)" [[25], p. 244].

Because participants had the opportunity to explore their own views and to hear alternative views expressed and defended, it is hard to explain the results of the present study as due to a lack of reflection, at least in comparison with other studies. In particular, the discussion group methodology allowed participants to engage with the task in a way that aids comprehension [26-30], that allowed them time to consider all of the alternatives carefully [31], that afforded them the opportunity to seek clarification of the task [32], and to construct considered views rather than simply self-report pre-existing preferences [[33], p. 47].

Elaborating on the final point, the deliberative approach has the potential to move participants towards 'strong evaluation' rather than 'weak evaluation'. Weak evaluation presupposes no more than that the subject be "a simple weigher of alternatives" [[34], p. 23]. In particular, it requires no more than the expression of personal preferences, and does not require subjects to go beyond the "self-interest" perspective [[14], pp. 43–47]. By contrast, strong evaluation presupposes that subjects have the ability not just to reflect upon alternatives, but also to reflect critically upon their own preferences, and to assess them as selfish, intolerant, generous, biased, and so on. Strong evaluation encourages subjects to adopt an 'other-regarding' or social perspective:

Groups and individuals thus enter the allocation decision process, not as simple weighters expressing irreducible preferences, but as strong evaluators, capable of recognizing the challenge that certain preferences make to our preconceptions of what health, human nature and the human community are or should be [[35], p. 251].

This does not eliminate the possibility that subjects might be confused about their own values or the matters of fact on which they are based. Nor does it eliminate the possibility of ethically questionable preferences being expressed. No procedure for resolving complex social problems can guarantee that. However, if techniques can be developed that facilitate strong evaluation rather than weak evaluation it lessens the likelihood of this happening, and provides a more secure foundation for public participation.

### (6) Comparison with the earlier Australian study

The present study differs from the earlier Australian study by Nord, Richardson et al. [6] in several ways. For example, although the earlier study involved a budget allocation exercise, those who had previously chosen to allocate to the cheapest patients or who had voted for a 'first come, first served' strategy were excluded. This meant that 47% of subjects did not complete the budget allocation exercise. Also, only five strategies were available in the earlier study, with the result that spending the same amount of money on both illnesses was not an explicit option, although this was the second most popular strategy in the present study.

Perhaps most importantly, the present study involved discussion groups, with an emphasis on reflection and deliberation; respondents chose their preferred method for allocating the hospital budget, for example, after in-depth discussion of the pros and cons of the various alternatives. In their paper, Nord, Richardson et al. [6] looked at a number of different reasons for downplaying the importance of costs (e.g. respondents may have anticipated their own emotional response if they were seriously ill, or may have felt a duty to those with a serious condition [6]), but they are the researchers' reasons rather than their subjects'.

Despite these differences, the same tendency for people to disregard costs in prioritising health care that was observed in the earlier study was also evident in the present study. In the decade that preceded 1995, Australia had implemented Medicare, a Commonwealth-funded health insurance scheme, providing all citizens free and universal access to health services. However, from 1995 to the current period the nation experienced a different social and political environment where government support for Medicare lessened, a greater emphasis on subsidised private health insurance emerged, and fiscal conservatism gradually became a bipartisan feature of political party health policy announcements [36]. Despite the differing social milieu, however, substantially the same attitude of Australians towards their public health system arose in the present study, along with further detail about the reasons for this.

## Conclusion

The importance of the study arises from the absolutely seminal role that costs play in standard economic evaluations of health programs and services, and the importance of public consultation. There have been a number of studies aimed at measuring social preferences for a variety of factors bearing on the allocation of resources; preferences for particular age groups [37-39], for the more severely ill [40-42], for the concentration and dispersion of health benefits [43-45]. However, these have all related to the benefits rather than the costs of health care. There have been relatively few studies looking directly at health costs, despite the critical role this plays in conventional evaluations of health services and programs. The few studies that have been conducted suggest that less importance is placed on costs by the general public than by economists. Confidence in these studies is considerably strengthened by the present study, which made strenuous efforts to ensure: (i) that participant's responses were reflective and informed; for example, that respondents had been exposed to, and had the opportunity of discussing, a range of options, and (ii) that they were very clear about the implications of their choices; that is, the opportunity costs were clear and explicit. Hopefully, as a result, the paper provides greater insight into why the public places less importance on costs than economists would consider appropriate.

## Competing interests

The authors declare that they have no competing interests.

## Authors' contributions

JM was one of the discussion group moderators, contributed to the development of the discussion protocol, was involved at all stages in the writing of the manuscript, and revised it in line with the Editor's and reviewers' suggestions. BS was one of the discussion group moderators, contributed to the development of the discussion protocol, and was involved at all stages in the writing of the manuscript. JR contributed to the development of the interview protocol, and contributed to the final drafts of the manuscript. RH contributed to the development of the interview protocol, and the final drafts of the manuscript.
